# Observations on an Obscure Point in Pathology

**Published:** 1846-06-01

**Authors:** 


*Observations on an Obscure Point in Pathology, by* N. S. Davis, M. D.

Dr. Davis communicates three cases of serious disorders, not easily located or named. In the first which he details, we suspect hypertrophy of heart to exist, in addition to other ailments. Cases of similar character must have fallen frequently under the notice of every practitioner. The nervous system is evidently chiefly involved. Dr. D. thinks the organic system more especially. There is a class of nervous disorders, infinitely varied in their particular and constantly mutating manifestations, but yet associated together by certain uniform phenomena, which are without any Nosological habitation and name. Will some one suggest a better title than *morbus neuropathicus?* We presume our practical readers will understand the class of affections referred to, without description; it would be a serious undertaking to attempt a history. The cases given by Dr. Davis illustrate some of the phases which it assumes. It is known by English writers as the *protean malady*, or *Hysteria; Dyspepsia* and *Spinal irritation* include a great number of specimens. We agree with Dr. D. that practitioners are too much given to neglect this class of ailments. Empirics, and venders of patent medicines thrive upon them.

